# Supplementary material for: Outcomes of hyperglycaemia in pregnancy in Africa: Systematic review and meta-analysis
Source: PLoS One. 2026 Mar 27;21(3):e0345743. doi: 10.1371/journal.pone.0345743 (PMC13029805; doi:10.1371/journal.pone.0345743)
Supplement: S1 Table — (DOCX) [file pone.0345743.s001.docx]

| S1 Table A. The Risk of Bias in Non-Randomised Studies – (ROBINS-I) assessment tool (n = 26) | | | | | | | |  |
| --- | --- | --- | --- | --- | --- | --- | --- | --- |
| \| **Study ID** \| \| --- \| | **1. Bias caused by confounding** | **2. Bias caused by selection of participants** | **3. Bias caused by classification of interventions** | **4. Bias caused by deviations from intended interventions** | **5. Attrition bias caused by missing data** | **6. Detection bias caused by measurement of outcomes** | **7. Reporting bias caused by selection of the reported results** | **Overall judgement** |
| Kheir et al 2012 | LOW | SERIOUS | NA | NA | No information | No information | LOW | **SERIOUS** |
| Magadla et al 2019 | LOW | SERIOUS | NA | NA | No information | No information | SERIOUS | **SERIOUS** |
| Mimouni-Zerguini et al 2009 | LOW | SERIOUS | NA | NA | No information | LOW | LOW | **MODERATE** |
| Mukona et al 2018 | LOW | SERIOUS | NA | NA | No information | LOW | LOW | **MODERATE** |
| Nakabuye et al 2017 | LOW | SERIOUS | NA | NA | No information | LOW | LOW | **MODERATE** |
| Odar et al 2004 | LOW | SERIOUS | NA | NA | No information | No information | No information | **SERIOUS** |
| Opara et al 2010 | LOW | SERIOUS | NA | NA | No information | No information | LOW | **MODERATE** |
| Ozumba et al 2004 | LOW | SERIOUS | NA | NA |  | No information | No information | **SERIOUS** |
| Soepnel et al 2019 | LOW | SERIOUS | NA | NA | No information | LOW | No information | **MODERATE** |
| Tandu-Umba et al 2012 | LOW | SERIOUS | NA | NA | No information | No information | No information | **SERIOUS** |
| van Zyl et al 2018 | LOW | SERIOUS | NA | NA | LOW | LOW | LOW | **MODERATE** |
| Chivese et al 2021 | LOW | LOW | NA | NA | LOW | LOW | LOW | **LOW** |
| Feleke et al 2020 | LOW | LOW | NA | NA | LOW | LOW | LOW | **LOW** |
| Muche et al 2020 | LOW | LOW | NA | NA | LOW | LOW | LOW | **LOW** |
| Abdelgadir M et al 2002 | LOW | SERIOUS | NA | NA | No information | No information | No information | **SERIOUS** |
| Bawah AT et al.2019 | LOW | SERIOUS | NA | NA | No information | No information | No information | **SERIOUS** |
| Bhorat I et al 2019 | LOW | LOW | NA | NA | No information | LOW | No information | **MODERATE** |
| Chivese T et al. 2019 | LOW | LOW | NA | NA | LOW | LOW | LOW | **LOW** |
| Coetzee A et al. 2018 | LOW | LOW | NA | NA | No information | LOW | No information | **MODERATE** |
| Dafallah S.E et al 2004 | LOW | SERIOUS | NA | NA | No information | No information | No information | **SERIOUS** |
| Daponte e Et al. 2009 | LOW | SERIOUS | NA | NA | No information | No information | No information | **SERIOUS** |
| Djagadou A et al. 2019 | LOW | SERIOUS | NA | NA | No information | No information | No information | **SERIOUS** |
| Ekpebegh E et al 2006 | LOW | SERIOUS | NA | NA | No information | No information | No information | **SERIOUS** |
| Bajrond E. et al. 2019 | LOW | LOW | NA | NA | No information | LOW | No information | **MODERATE** |
| Huddle KR et.al 2005 | LOW | SERIOUS | NA | NA | No information | No information | No information | **SERIOUS** |
| John CO et.al 2015 | LOW | SERIOUS | NA | NA | No information | No information | No information | **SERIOUS** |
| Malaza 2023 | LOW | SERIOUS | NA | NA | No information | LOW | No information | **SERIOUS** |
| Burodo et al2023 | LOW | SERIOUS | NA | NA | No information | LOW | No information | **SERIOUS** |
| S1 Table B. Risk of bias for included RCTs (n = 2) | | | | | | | | |

| Authors, year | Selection bias, random sequence generation | Selection bias (allocation concealment) | Detection bias, blinding (outcome assessment) | Attrition bias, incomplete outcome data | Reporting bias, selective reporting | Performance bias, blinding (participants and personnel) |
| --- | --- | --- | --- | --- | --- | --- |
| Maged et al 2016 | NCR | NCR | NCR | Low | NCR | High |
| Utz et al 2018 | Low | Low | low | Low | Low | Low |

**NA= Not Applicable**

***NCR:* No Clear Risk**

|  |
| --- |
