# Supplementary material for: Outcomes of hyperglycaemia in pregnancy in Africa: Systematic review and meta-analysis
Source: PLoS One. 2026 Mar 27;21(3):e0345743. doi: 10.1371/journal.pone.0345743 (PMC13029805; doi:10.1371/journal.pone.0345743)
Supplement: S2 Table — (DOCX) [file pone.0345743.s002.docx]

| S2 Table. Assessment of quality of evidence (GRADE) in the included studies (n = 30) | | | | | | | |
| --- | --- | --- | --- | --- | --- | --- | --- |
| **Study ID** | **Study Design** | **Study Limitation** | **Inconsistency** | **Indirectness** | **Imprecision** | **Bias** | **Overall Quality** |
| Kheir et al 2012 | Observational prospective | **X** | **√** | **√** | **X** | **X** | **++** |
| Magadla et al 2019 | Retrospective medical record review | **X** | **√** | **X** | **X** | **X** | **+** |
| Maged et al 2016 | Randomized control trial - control included | **√** | **√** | **√** | **√** | **X** | **++++** |
| Mimouni-Zerguini et al **2009** | Prospective | **X** | **√** | **√** | **√** | **X** | **+++** |
| Mukona et al **2018** | Prospective | **X** | **√** | **√** | **√** | **X** | **+++** |
| Nakabuye et al **2017** | Prospective | **X** | **√** | **√** | **√** | **X** | **+++** |
| Odar et al **2004** | Prospective | **X** | **√** | **√** | **√** | **X** | **+++** |
| Opara et al **2010** | Prospective | **X** | **√** | **√** | **√** | **X** | **+++** |
| Ozumba et al **2004** | Retrospective medical record review | **X** | **√** | **X** | **X** | **X** | **+** |
| Soepnel et al **2019** | Retrospective medical record review | **X** | **√** | **X** | **X** | **X** | **+** |
| Tandu-Umba et al **2012** | Prospective | **X** | **√** | **√** | **√** | **X** | **+++** |
| Utz et al 208 | Randomized control trial (screening and detection) | **√** | **√** | **√** | **√** | **X** | **++++** |
| van Zyl et al **2018** | Retrospective descriptive | **X** | **√** | **√** | **X** | **X** | **++** |
| Chivese et al **2021** | Prospective | **X** | **√** | **√** | **√** | **X** | **+++** |
| Feleke et al **2020** | retrospective and prospective cohort |  |  |  |  |  |  |
| Muche et al **2020** | prospective cohort | **√** | **√** | **√** | **√** | **X** | **++++** |
| Abdelgadir M et al **2002** | Prospective, case control | **X** | **√** | **√** | **X** | **X** | **++** |
| Bawah AT et al.**2019** | Retrospective case control | **X** | **√** | **X** | **√** | **X** | **++** |
| Bhorat I et al **2019** | Prospective, cross sectional study | **X** | **√** | **√** | **√** | **X** | **+++** |
| Chivese T et al. **2019** | Cross sectional | **X** | **√** | **√** | **√** | **X** | **+++** |
| Coetzee A et al. **2018** | Cross-sectional | **X** | **√** | **√** | **√** | **X** | **+++** |
| Dafallah S.E et al **2004** | Prospective cohort | **X** | **√** | **√** | **X** | **X** | **++** |
| Daponte e Et al. **2009** | Prospective case control | **X** | **√** | **√** | **X** | **X** | **++** |
| Djagadou A et al. **2019** | Retrospective audit | **X** | **√** | **X** | **X** | **X** | **+** |
| Ekpebegh E et al **2006** | Retrospective audit | X | √ | X | **X** | X | + |
| Bajrond E. et al. **2019** | Retrospective audit | X | √ | X | **X** | X | + |
| Huddle KR et.al **2005** | Retrospective, cross-sectional | X | √ | X | **√** | X | ++ |
| John CO et.al **2015** | Retrospective analysis | X | √ | X | **X** | X | + |

**√ = no serious limitations; X = serious limitations, for overall quality of evidence: + = very low; ++ = low; +++ = moderate; ++++ = high;**

**RCT: Randomize Control Trials.**
