# Supplementary material for: Outcomes of hyperglycaemia in pregnancy in Africa: Systematic review and meta-analysis
Source: PLoS One. 2026 Mar 27;21(3):e0345743. doi: 10.1371/journal.pone.0345743 (PMC13029805; doi:10.1371/journal.pone.0345743)

Supplementary subgroup analyses

Caesarian section prevalence by country

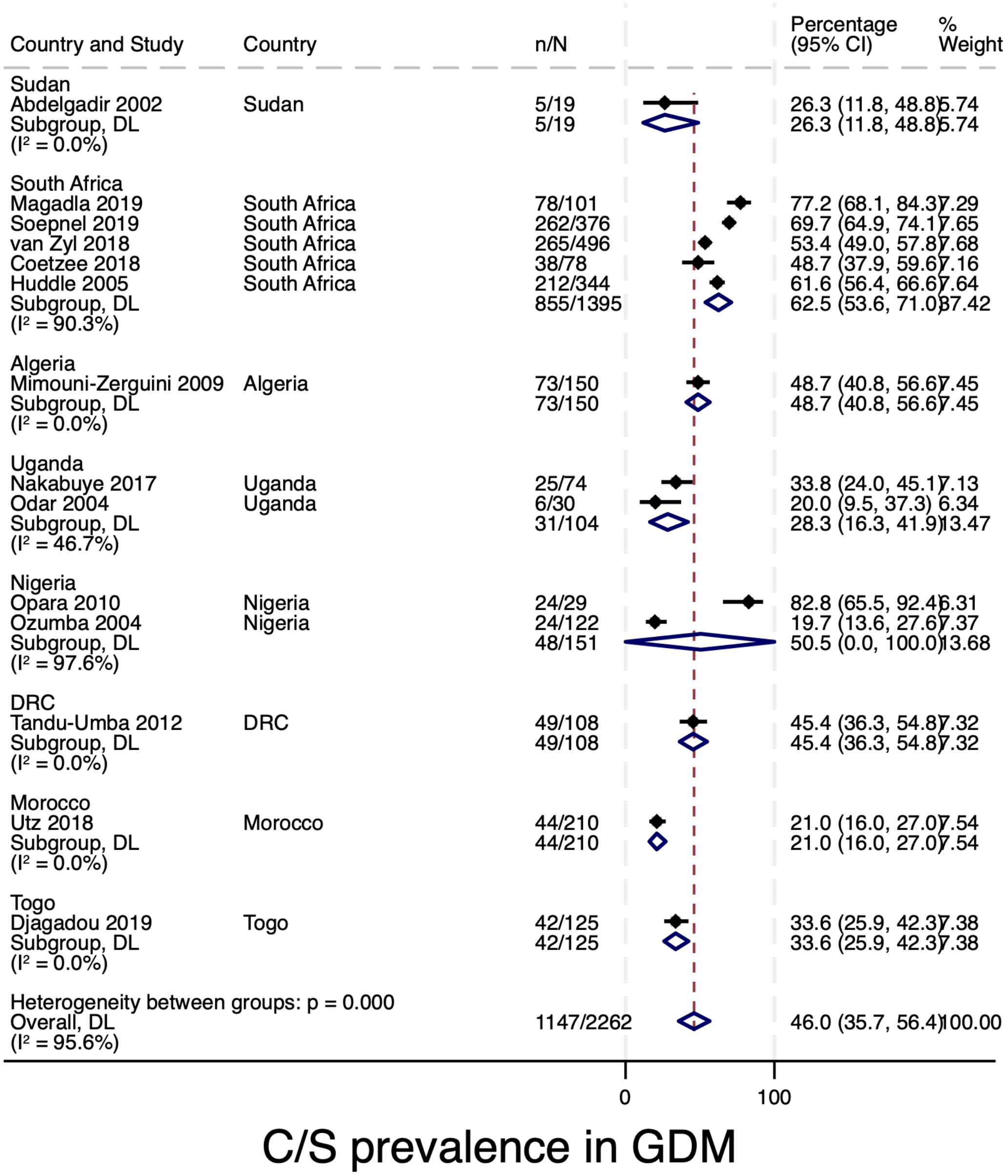

NOTE: Weights and between-subgroup heterogeneity test are from random-effects model

Macrosomia by country

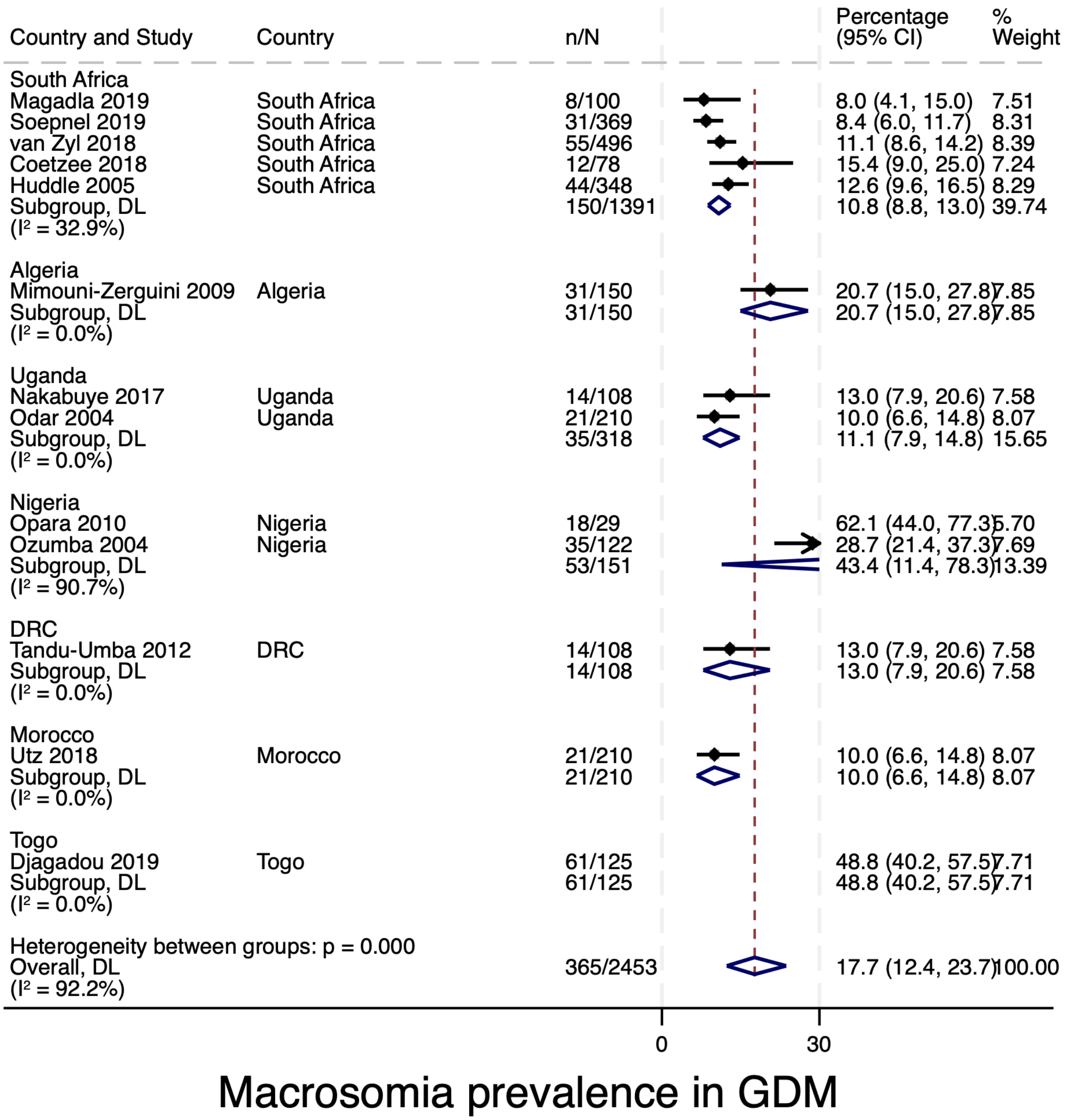

NOTE: Weights and between-subgroup heterogeneity test are from random-effects model

Congenital Malformations by country

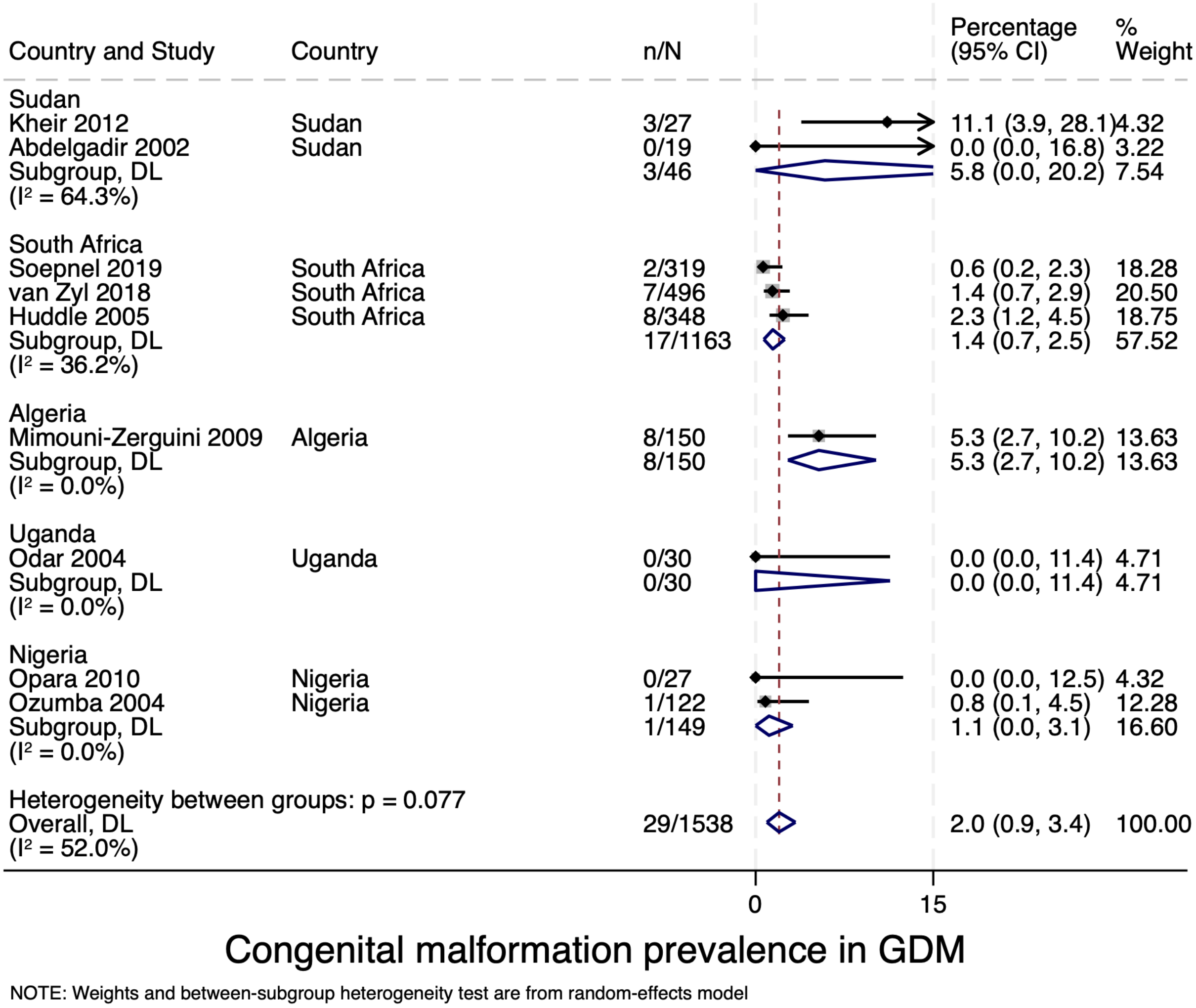

Neonatal hypoglycemia by country

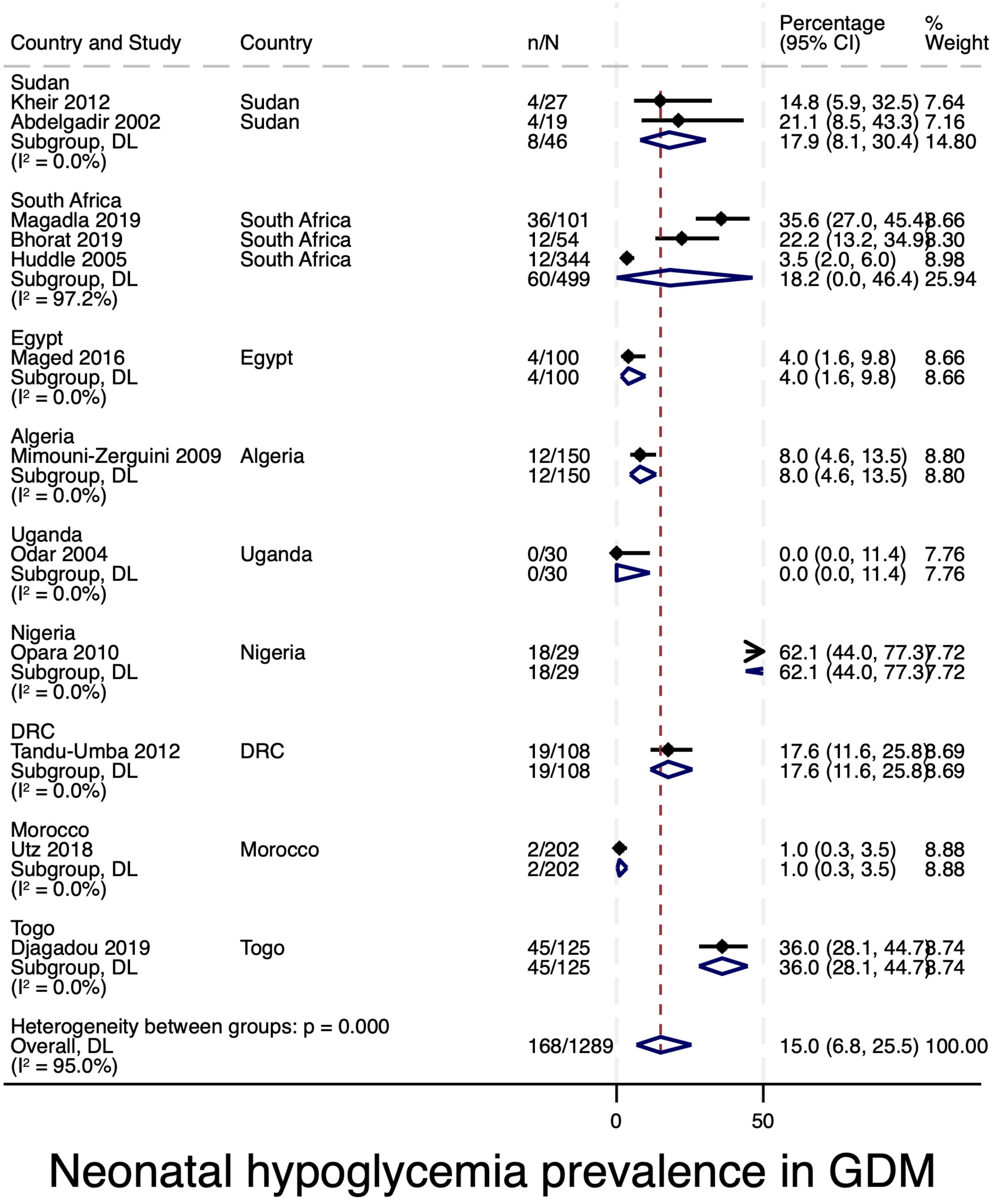

NOTE: Weights and between-subgroup heterogeneity test are from random-effects model

Neonatal death by country

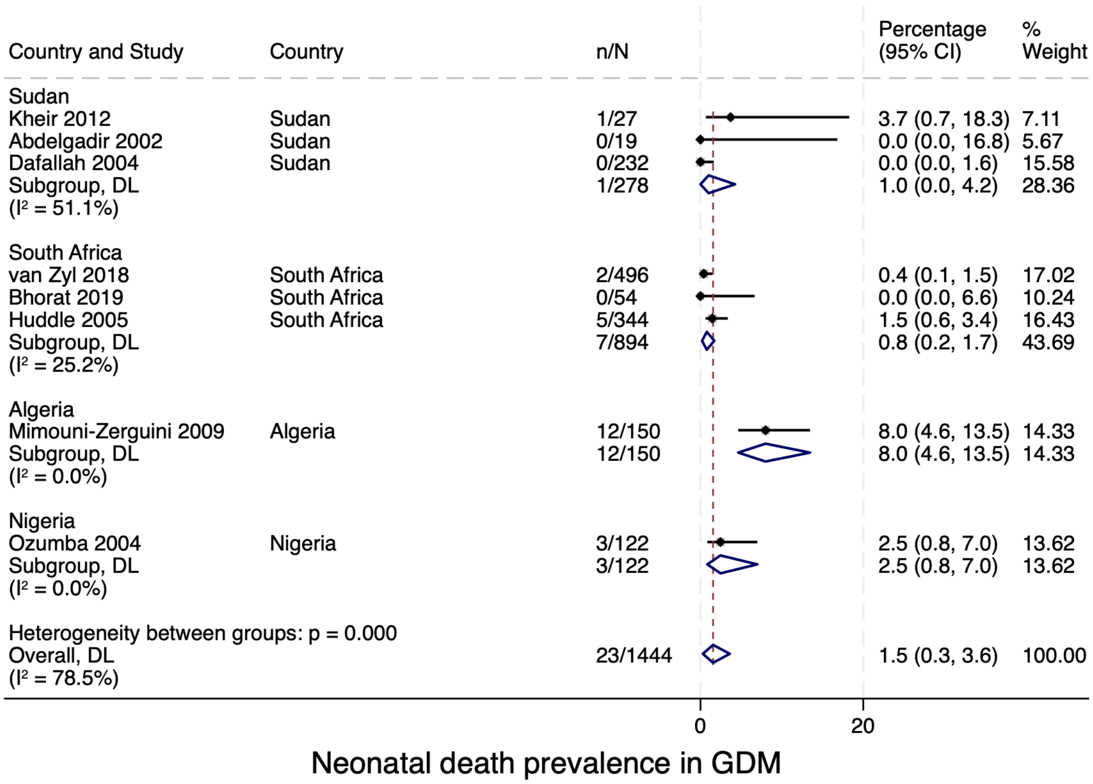

NOTE: Weights and between-subgroup heterogeneity test are from random-effects model

Stillbirth by country

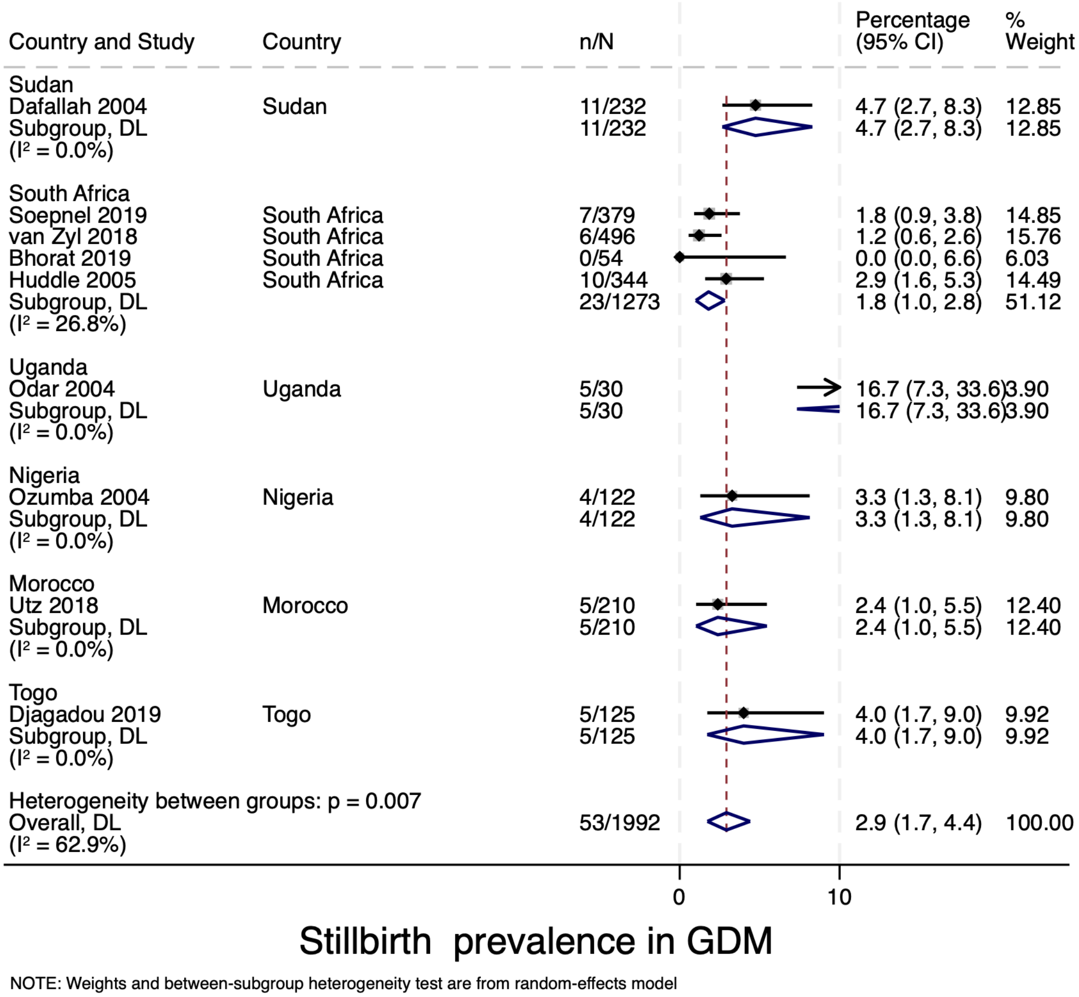

Preterm prevalence by country

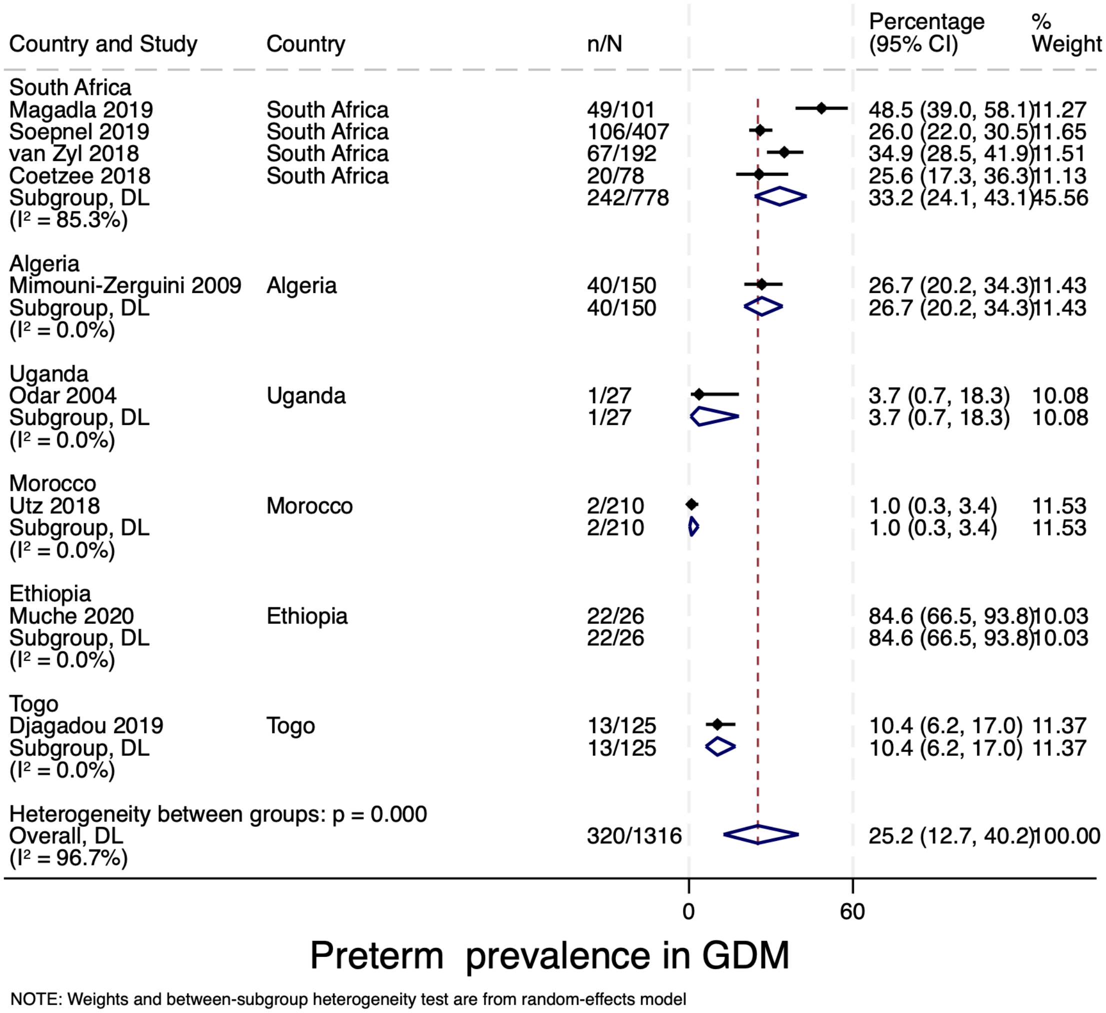

Supplement: S1 File — (PDF) [file pone.0345743.s003.pdf]
