# Supplementary material for: Outcomes of hyperglycaemia in pregnancy in Africa: Systematic review and meta-analysis
Source: PLoS One. 2026 Mar 27;21(3):e0345743. doi: 10.1371/journal.pone.0345743 (PMC13029805; doi:10.1371/journal.pone.0345743)
Supplement: S3 File — (PDF) [file pone.0345743.s005.pdf]

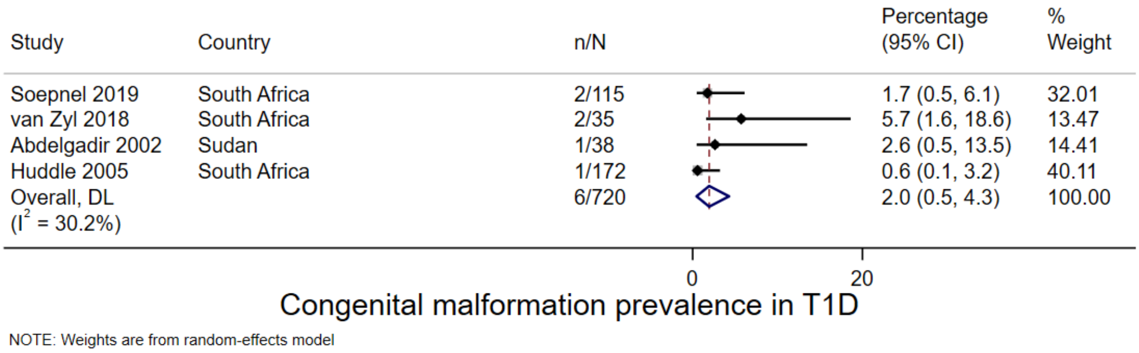
Study
Country
n/N
Percentage (95% CI)
% Weight
Soepnel 2019 South Africa 2/115 1.7 (0.5, 6.1) 32.01
van Zyl 2018 South Africa 2/35 5.7 (1.6, 18.6) 13.47
Abdelgadir 2002 Sudan 1/38 2.6 (0.5, 13.5) 14.41
Huddle 2005 South Africa 1/172 0.6 (0.1, 3.2) 40.11
Overall, DL 6/720 2.0 (0.5, 4.3) 100.00
(I² = 30.2%)
0
20
Congenital malformation prevalence in T1D
NOTE: Weights are from random-effects model

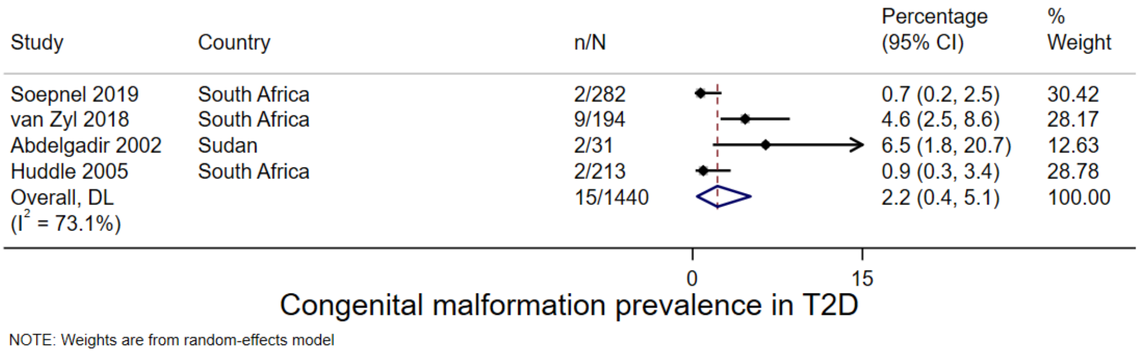
Study
Country
n/N
Percentage (95% CI)
% Weight
Soepnel 2019 South Africa 2/282 0.7 (0.2, 2.5) 30.42
van Zyl 2018 South Africa 9/194 4.6 (2.5, 8.6) 28.17
Abdelgadir 2002 Sudan 2/31 6.5 (1.8, 20.7) 12.63
Huddle 2005 South Africa 2/213 0.9 (0.3, 3.4) 28.78
Overall, DL 15/1440 2.2 (0.4, 5.1) 100.00
(I² = 73.1%)
0
15
Congenital malformation prevalence in T2D
NOTE: Weights are from random-effects model

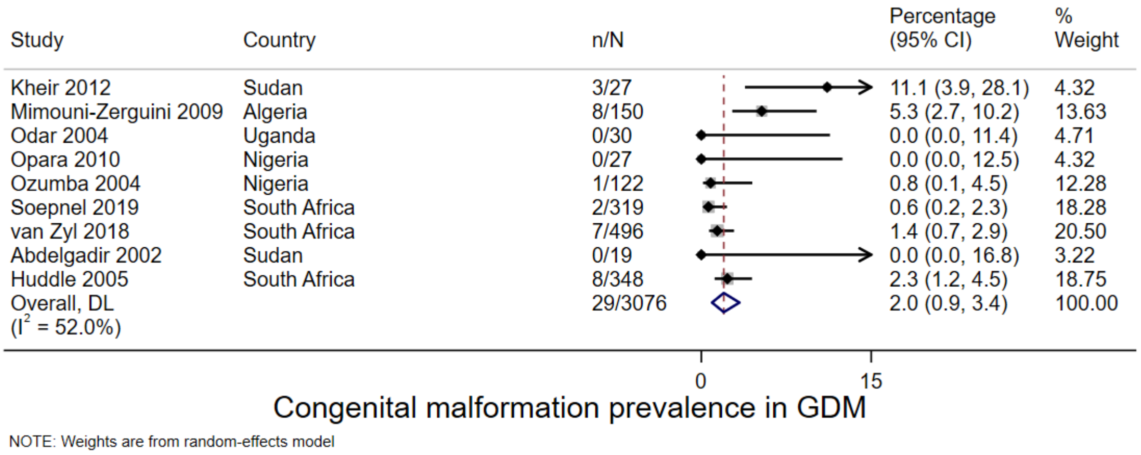
Study
Country
n/N
Percentage (95% CI)
% Weight
Kheir 2012 Sudan 3/27 11.1 (3.9, 28.1) 4.32
Mimouni-Zerguini 2009 Algeria 8/150 5.3 (2.7, 10.2) 13.63
Odar 2004 Uganda 0/30 0.0 (0.0, 11.4) 4.71
Opara 2010 Nigeria 0/27 0.0 (0.0, 12.5) 4.32
Ozumba 2004 Nigeria 1/122 0.8 (0.1, 4.5) 12.28
Soepnel 2019 South Africa 2/319 0.6 (0.2, 2.3) 18.28
van Zyl 2018 South Africa 7/496 1.4 (0.7, 2.9) 20.50
Abdelgadir 2002 Sudan 0/19 0.0 (0.0, 16.8) 3.22
Huddle 2005 South Africa 8/348 2.3 (1.2, 4.5) 18.75
Overall, DL 29/3076 2.0 (0.9, 3.4) 100.00
(I² = 52.0%)
0
15
Congenital malformation prevalence in GDM
NOTE: Weights are from random-effects model

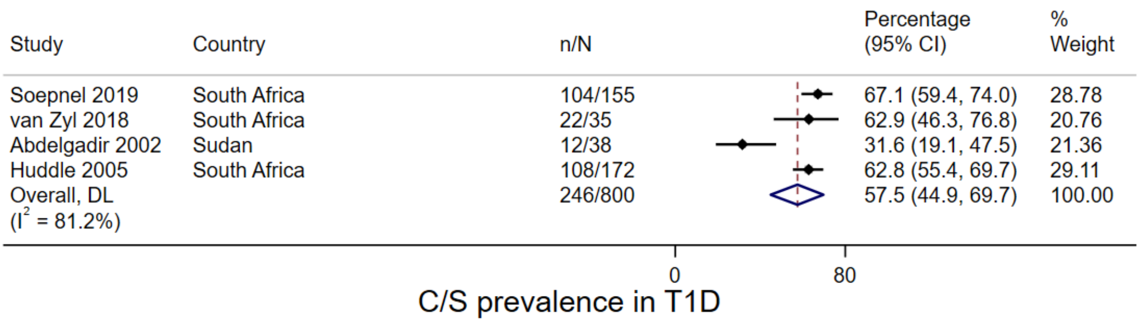

NOTE: Weights are from random-effects model

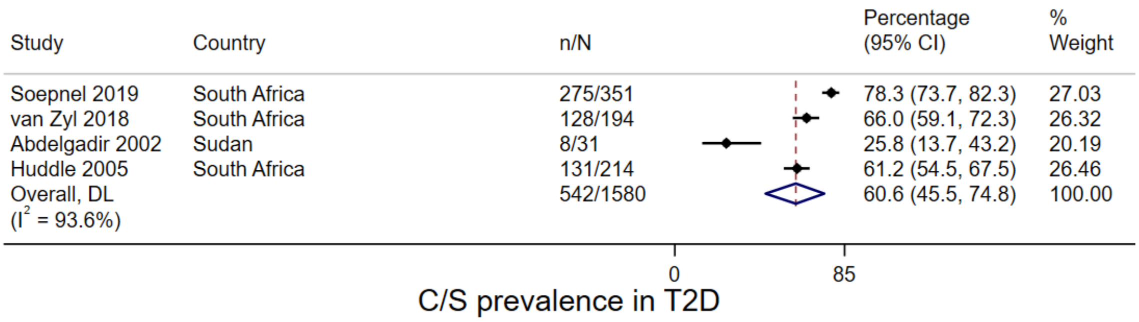

NOTE: Weights are from random-effects model

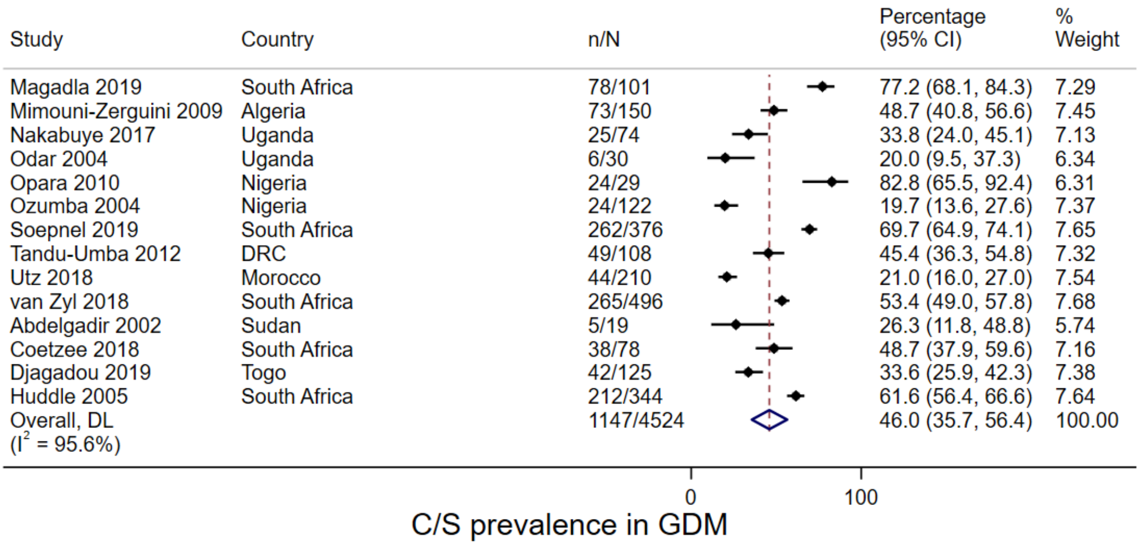

NOTE: Weights are from random-effects model

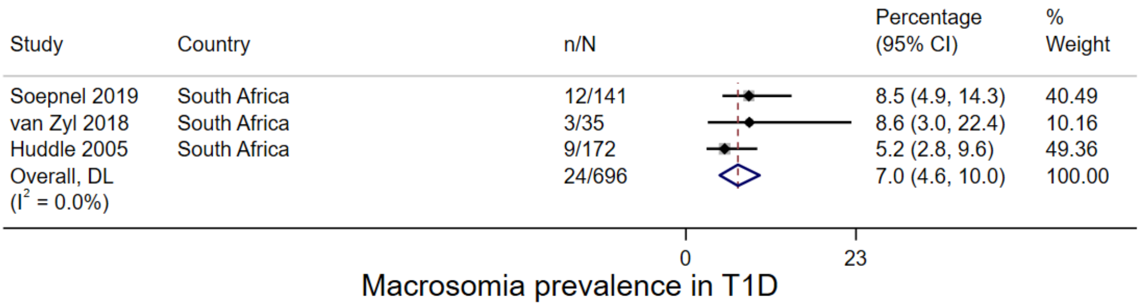

NOTE: Weights are from random-effects model

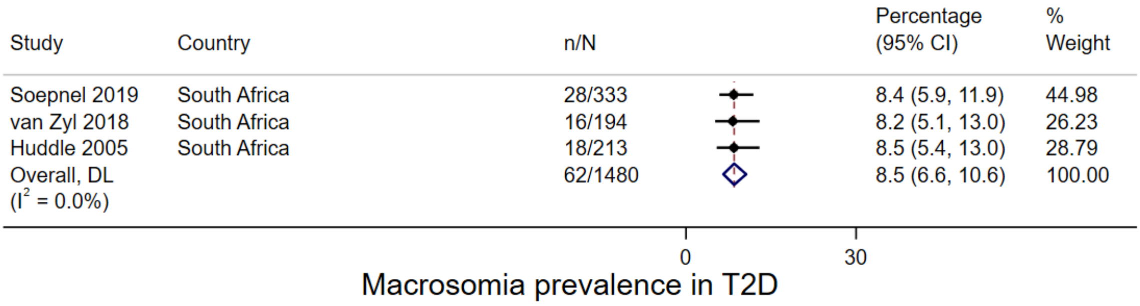

NOTE: Weights are from random-effects model

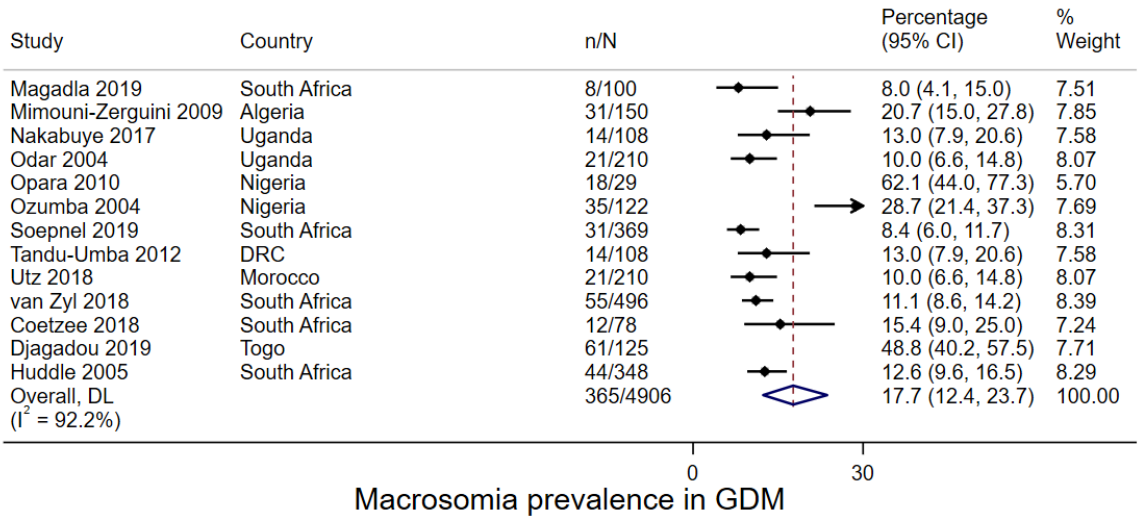

NOTE: Weights are from random-effects model

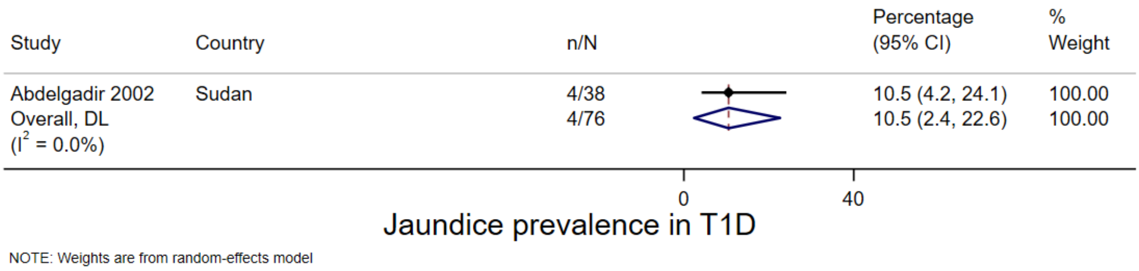
Study
Country
n/N
Percentage (95% CI)
% Weight
Abdelgadir 2002 Sudan 4/38 10.5 (4.2, 24.1) 100.00
Overall, DL 4/76 10.5 (2.4, 22.6) 100.00
(I^2 = 0.0%)
0
40
Jaundice prevalence in T1D
NOTE: Weights are from random-effects model

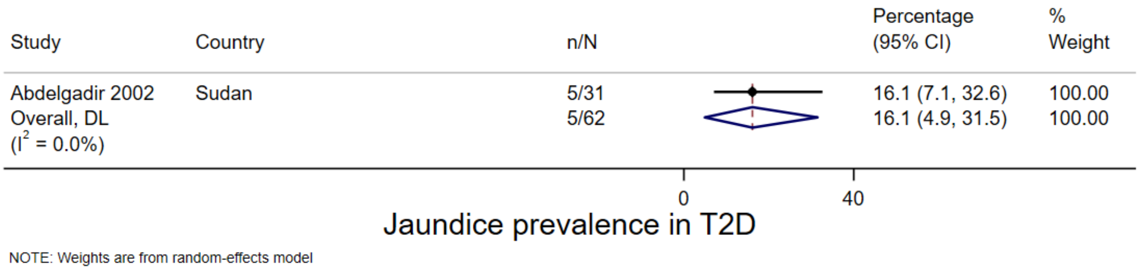
Study
Country
n/N
Percentage (95% CI)
% Weight
Abdelgadir 2002 Sudan 5/31 16.1 (7.1, 32.6) 100.00
Overall, DL 5/62 16.1 (4.9, 31.5) 100.00
(I^2 = 0.0%)
0
40
Jaundice prevalence in T2D
NOTE: Weights are from random-effects model

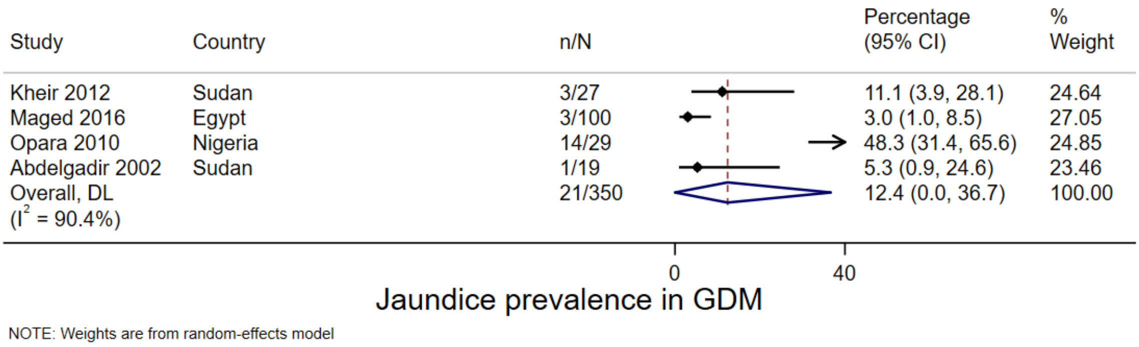
Study
Country
n/N
Percentage (95% CI)
% Weight
Kheir 2012 Sudan 3/27 11.1 (3.9, 28.1) 24.64
Maged 2016 Egypt 3/100 3.0 (1.0, 8.5) 27.05
Opara 2010 Nigeria 14/29 48.3 (31.4, 65.6) 24.85
Abdelgadir 2002 Sudan 1/19 5.3 (0.9, 24.6) 23.46
Overall, DL 21/350 12.4 (0.0, 36.7) 100.00
(I^2 = 90.4%)
0
40
Jaundice prevalence in GDM
NOTE: Weights are from random-effects model

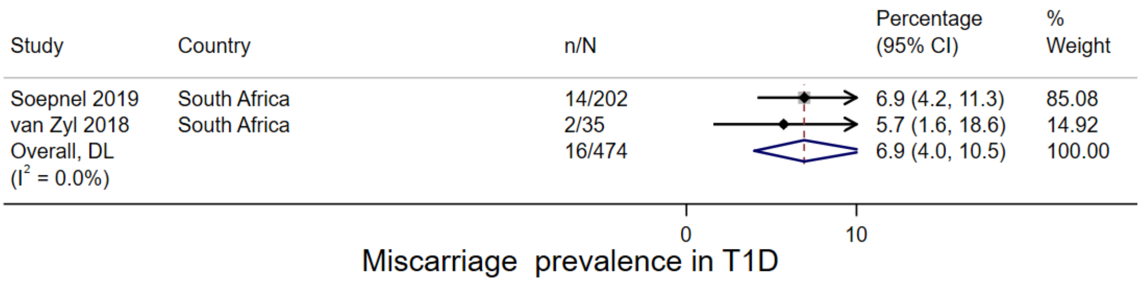

NOTE: Weights are from random-effects model

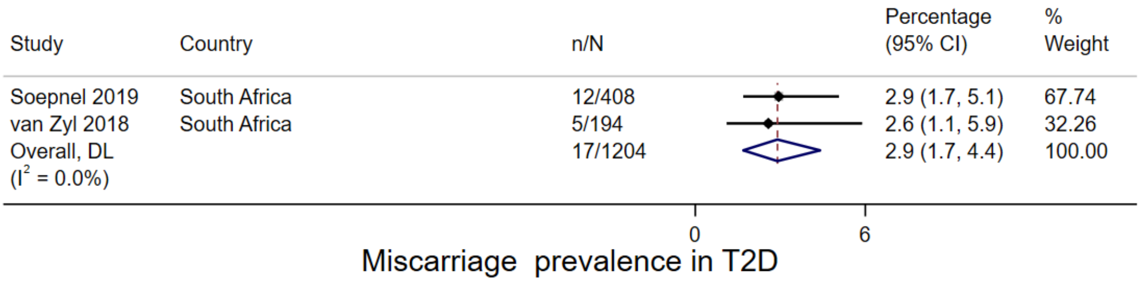

NOTE: Weights are from random-effects model

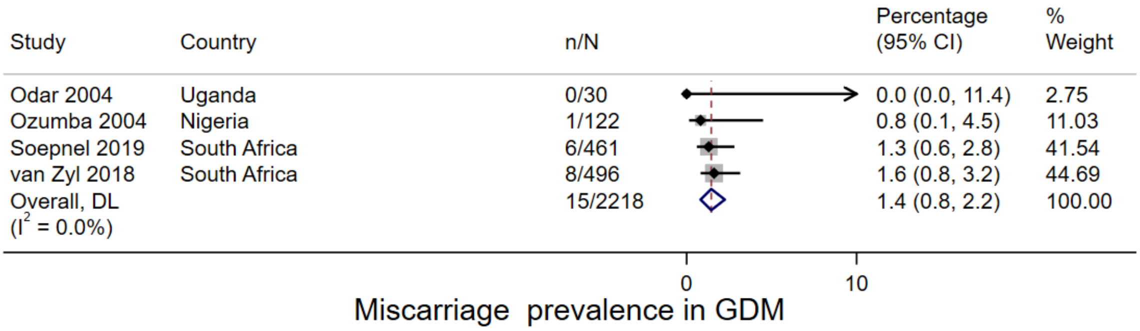

NOTE: Weights are from random-effects model

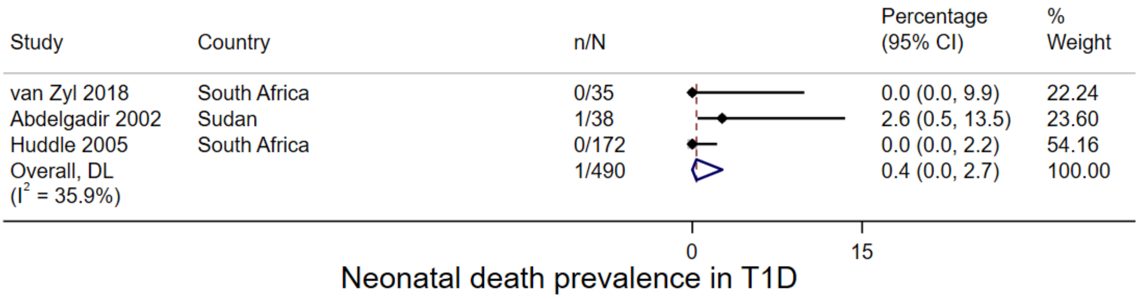

NOTE: Weights are from random-effects model

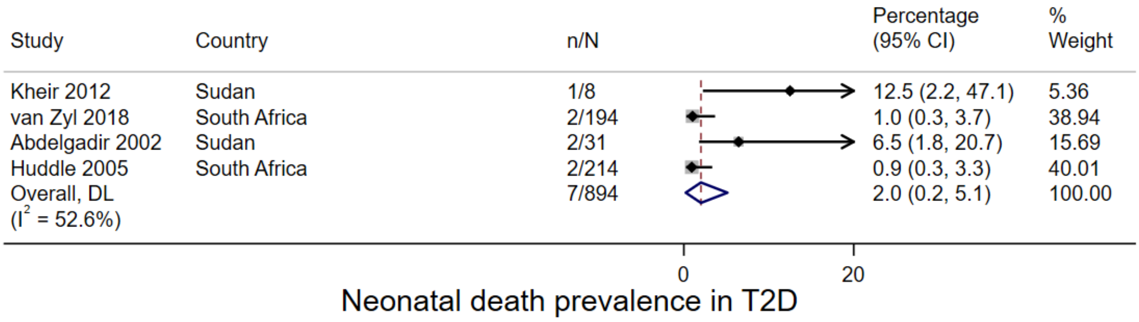

NOTE: Weights are from random-effects model

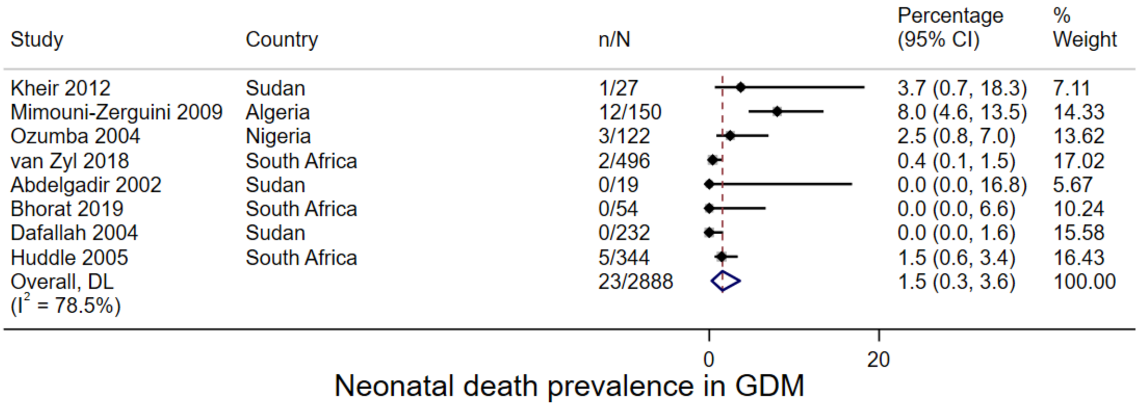

NOTE: Weights are from random-effects model

Study	Country	n/N	Percentage (95% CI)	% Weight
Abdelgadir 2002	Sudan	16/38	42.1 (27.9, 57.8)	48.29
Huddle 2005	South Africa	17/172	9.9 (6.3, 15.3)	51.71
Overall, DL (I^2 = 94.6%)		33/420	20.2 (0.0, 61.4)	100.00

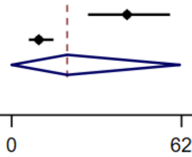

Neonatal hypoglycemia prevalence in T1D

NOTE: Weights are from random-effects model

Study	Country	n/N	Percentage (95% CI)	% Weight
Abdelgadir 2002	Sudan	7/31	22.6 (11.4, 39.8)	43.28
Huddle 2005	South Africa	16/214	7.5 (4.7, 11.8)	56.72
Overall, DL (I^2 = 81.9%)		23/490	11.9 (0.7, 30.6)	100.00

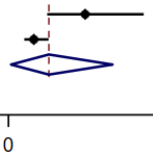

Neonatal hypoglycemia prevalence in T2D

NOTE: Weights are from random-effects model

Study	Country	n/N	Percentage (95% CI)	% Weight
Kheir 2012	Sudan	4/27	14.8 (5.9, 32.5)	7.64
Magadla 2019	South Africa	36/101	35.6 (27.0, 45.4)	8.66
Maged 2016	Egypt	4/100	4.0 (1.6, 9.8)	8.66
Mimouni-Zerguini 2009	Algeria	12/150	8.0 (4.6, 13.5)	8.80
Odar 2004	Uganda	0/30	0.0 (0.0, 11.4)	7.76
Opara 2010	Nigeria	18/29	62.1 (44.0, 77.3)	7.72
Tandu-Umba 2012	DRC	19/108	17.6 (11.6, 25.8)	8.69
Utz 2018	Morocco	2/202	1.0 (0.3, 3.5)	8.88
Abdelgadir 2002	Sudan	4/19	21.1 (8.5, 43.3)	7.16
Bhorat 2019	South Africa	12/54	22.2 (13.2, 34.9)	8.30
Djagadou 2019	Togo	45/125	36.0 (28.1, 44.7)	8.74
Huddle 2005	South Africa	12/344	3.5 (2.0, 6.0)	8.98
Overall, DL (I^2 = 95.0%)		168/2578	15.0 (6.8, 25.5)	100.00

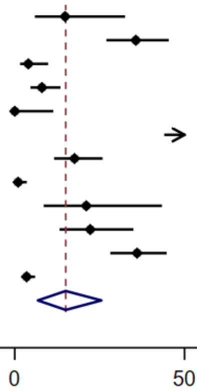

Neonatal hypoglycemia prevalence in GDM

NOTE: Weights are from random-effects model

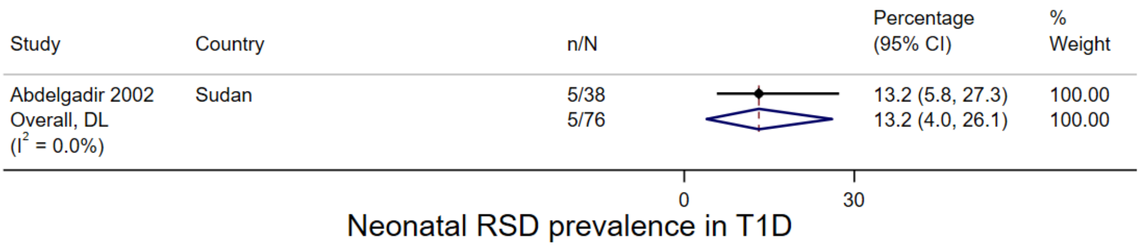

NOTE: Weights are from random-effects model

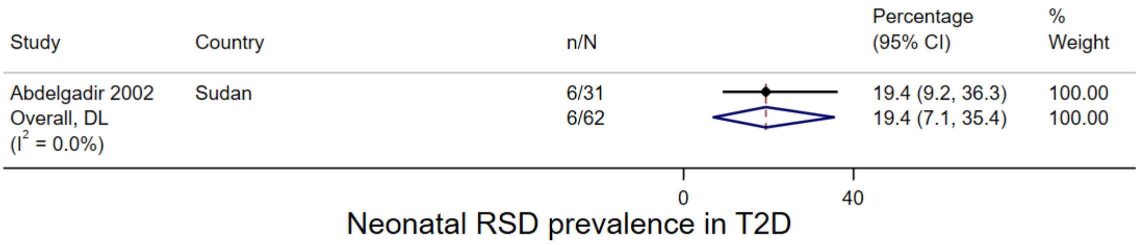

NOTE: Weights are from random-effects model

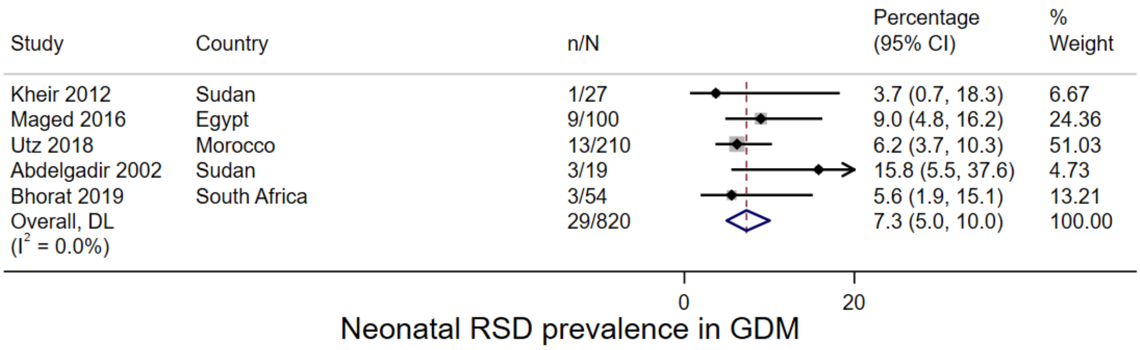

NOTE: Weights are from random-effects model

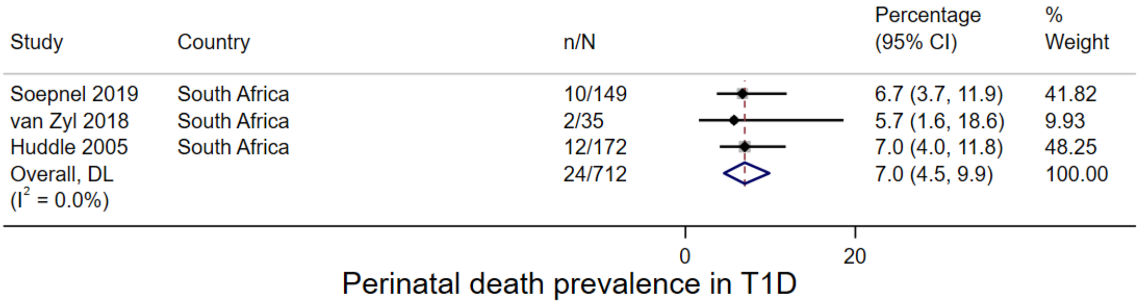

NOTE: Weights are from random-effects model

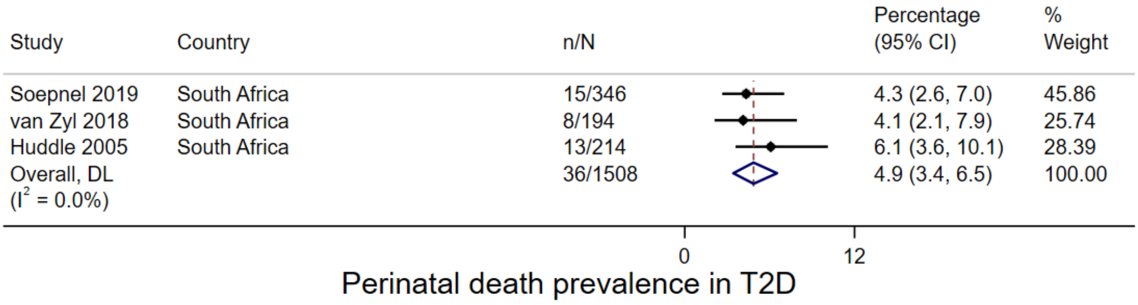

NOTE: Weights are from random-effects model

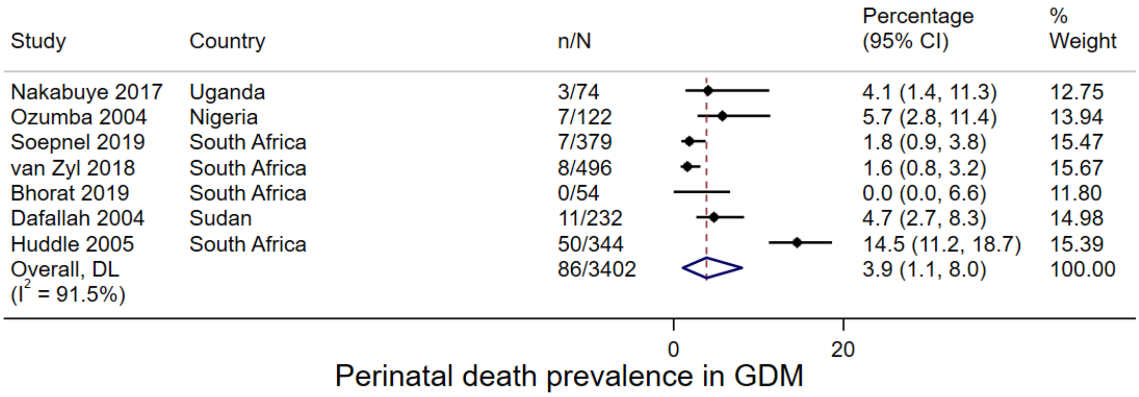

NOTE: Weights are from random-effects model

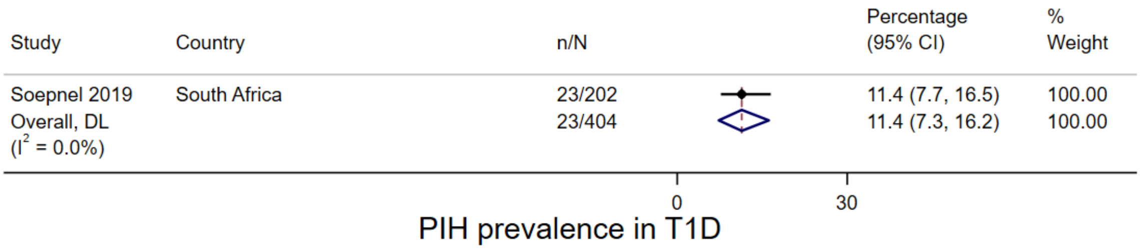

NOTE: Weights are from random-effects model

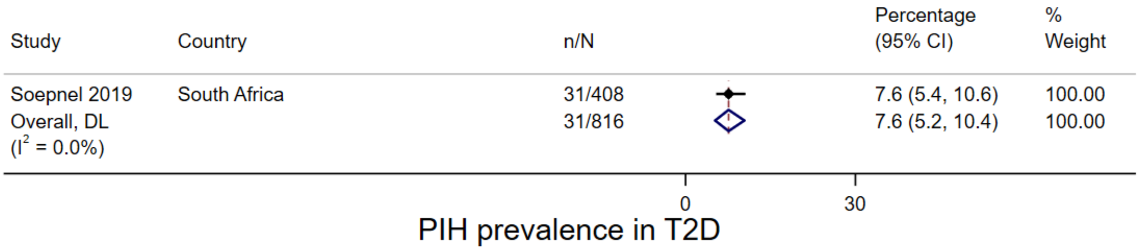

NOTE: Weights are from random-effects model

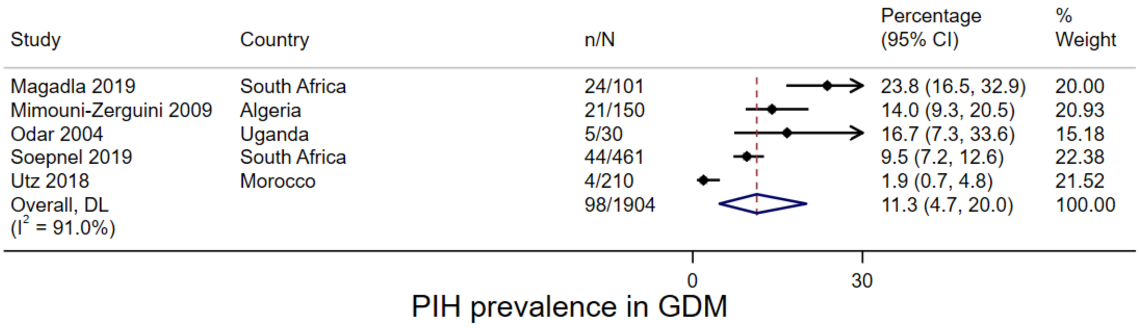

NOTE: Weights are from random-effects model

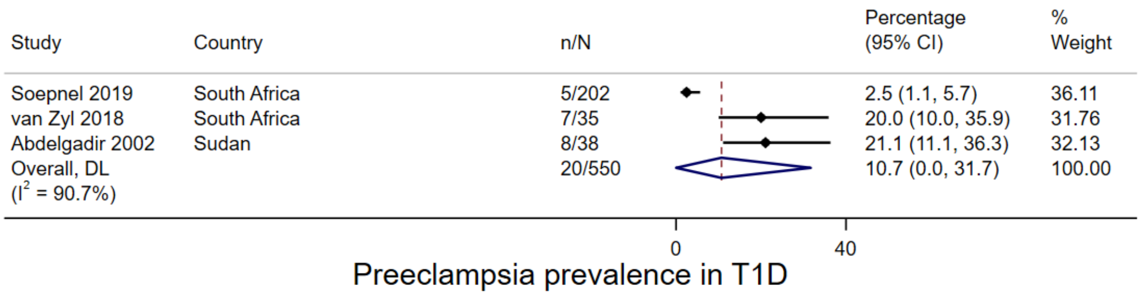

NOTE: Weights are from random-effects model

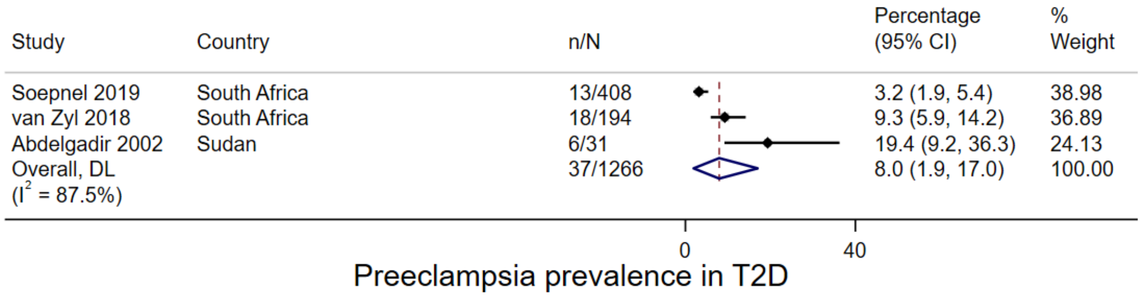

NOTE: Weights are from random-effects model

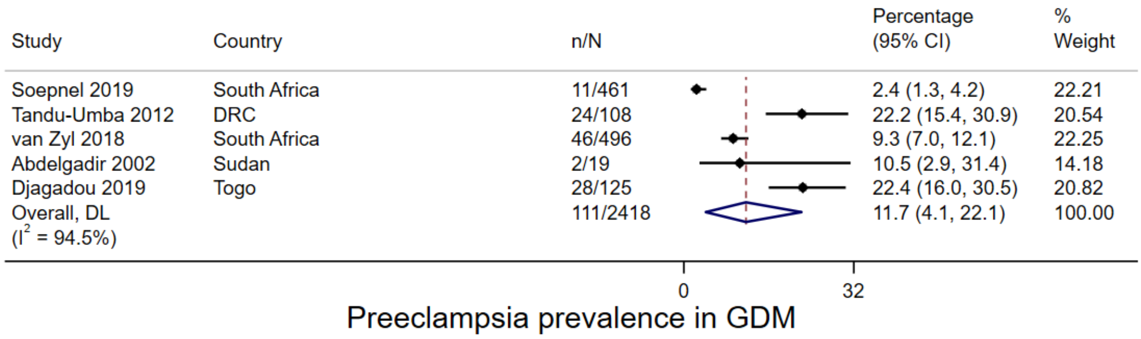

NOTE: Weights are from random-effects model

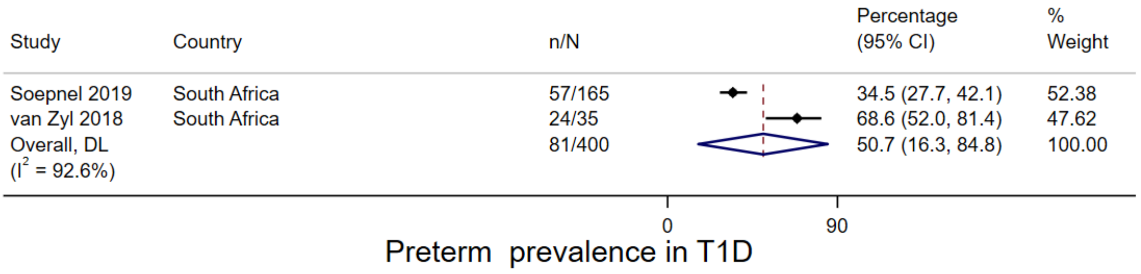

NOTE: Weights are from random-effects model

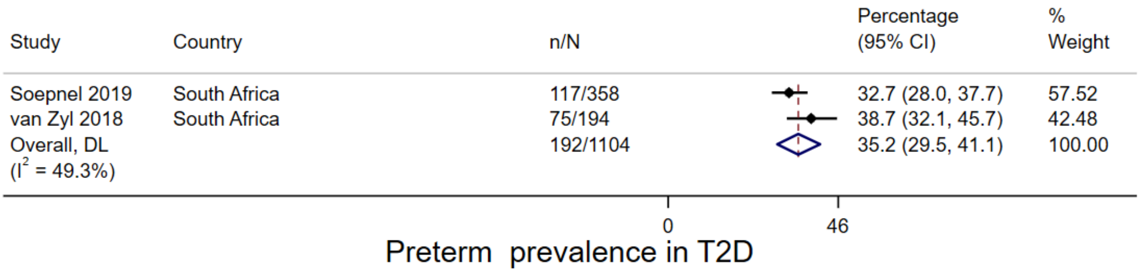

NOTE: Weights are from random-effects model

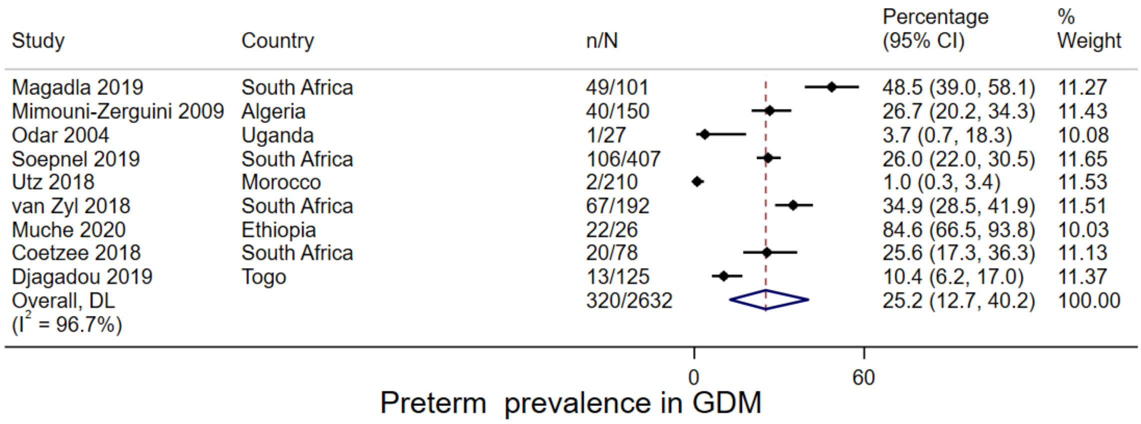

NOTE: Weights are from random-effects model

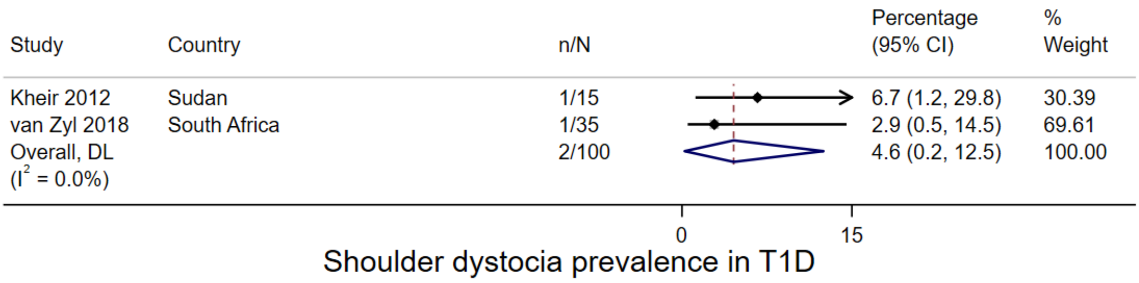

NOTE: Weights are from random-effects model

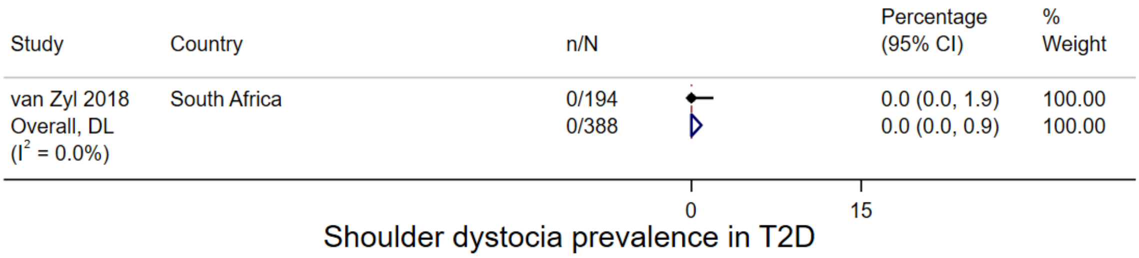

NOTE: Weights are from random-effects model

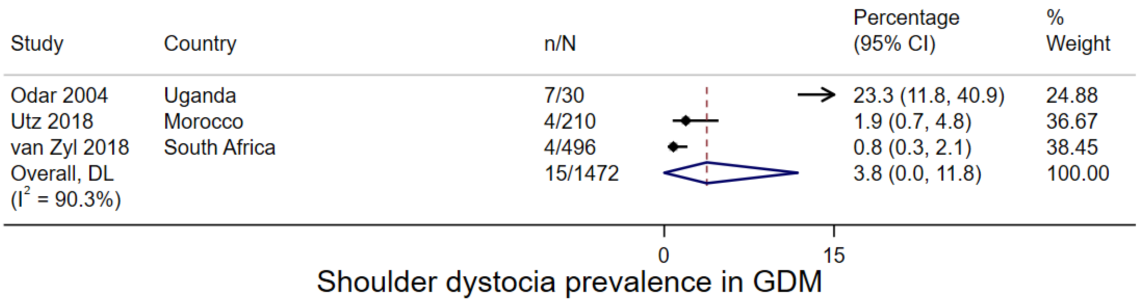

NOTE: Weights are from random-effects model

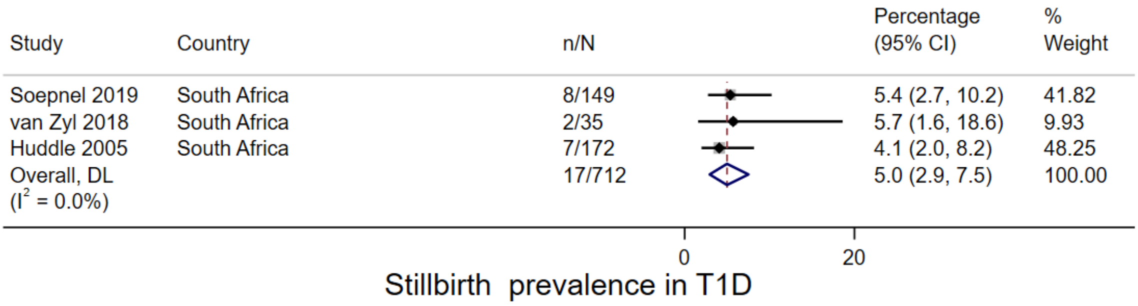

NOTE: Weights are from random-effects model

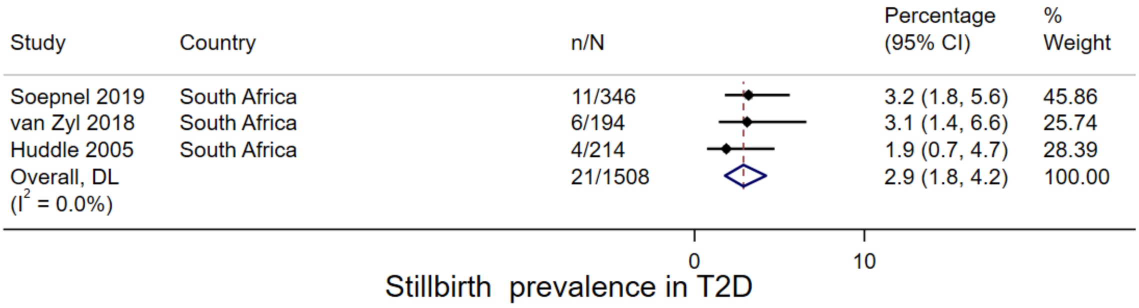

NOTE: Weights are from random-effects model

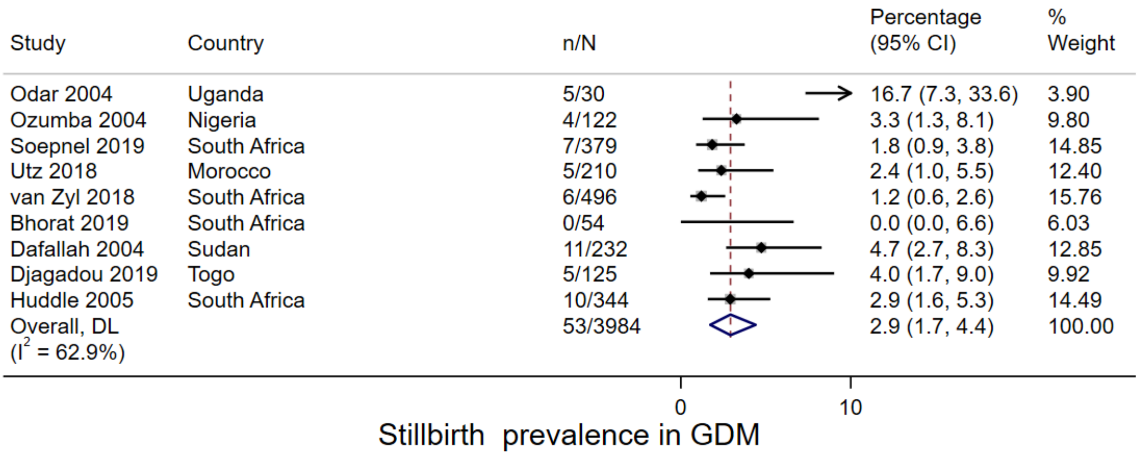

NOTE: Weights are from random-effects model

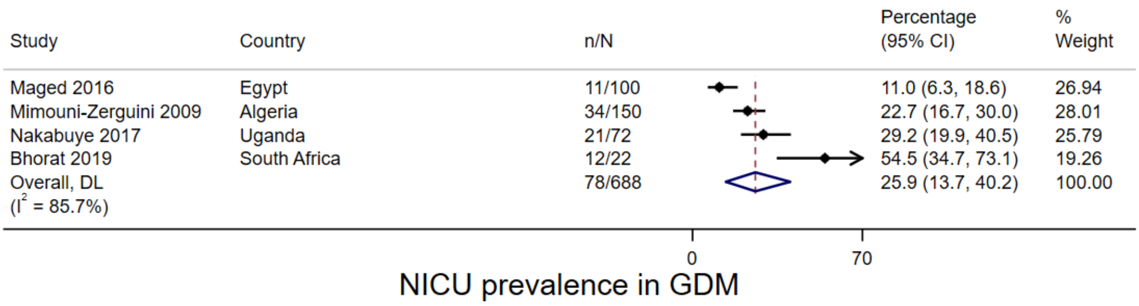

NOTE: Weights are from random-effects model
