# Supplementary material for: Outcomes of hyperglycaemia in pregnancy in Africa: Systematic review and meta-analysis
Source: PLoS One. 2026 Mar 27;21(3):e0345743. doi: 10.1371/journal.pone.0345743 (PMC13029805; doi:10.1371/journal.pone.0345743)
Supplement: Attachment — Submitted filename: Responses_to_Reviewers_auresp_2.docx [file pone.0345743.s009.docx]

**Responses to Reviewers' comments**

Additional Editor Comments:

Although the authors have revised the manuscript following the initial reviewers´ comments, the revision on the high heterogeneity found across the studies were poorly addressed in the current version. This point needs major improvement and revision not only on Discussion section but also in Abstract, Limitations in the Scientific context section, and presentation of the results.

Author’s response: We thank the reviewer for highlighting the need to improve and revise the high heterogeneity observed across the included studies in our review. We have substantially revised the manuscript to address this point. Find revisions in Lines 68 – 79, 86 – 88; 132 - 134; 293 – 294; 331, 363 – 365, 367 – 372, 387 – 388, 392 – 394, 404 – 413; and lines 438 – 439, 596 – 598. These revisions clarify how heterogeneity impacts the interpretation of our findings and strengthen the review's transparency.
